# Crystal structure of 5-chloro-1,3-bis­[2-(2-oxo-1,3-oxazolidin-3-yl)eth­yl]-1*H*-benz­imidazol-2(3*H*)-one

**DOI:** 10.1107/S2056989015016102

**Published:** 2015-09-12

**Authors:** Kaoutar Bouayad, Youssef Kandri Rodi, Youness Ouzidan, El Mokhtar Essassi, Mohamed Saadi, Lahcen El Ammari

**Affiliations:** a Laboratoire de Chimie de la Matière condensée, Faculté des Sciences et Techniques, Université Sidi Mohamed Ben Abdallah, Fès, Morocco; b Laboratoire de Chimie Organique Appliquée, Université Sidi Mohamed Ben Abdallah, Faculté des Sciences et Techniques, Route d’Immouzzer, BP 2202, Fès, Morocco; c Laboratoire de Chimie Organique Hétérocyclique, URAC 21, Pôle de Compétences Pharmacochimie, Université Mohammed V, BP 1014 Avenue Ibn Batouta, Rabat, Morocco; d Laboratoire de Chimie du Solide Appliquée, Faculté des Sciences, Université Mohammed V, Avenue Ibn Battouta, BP 1014, Rabat, Morocco

**Keywords:** crystal structure, benzimidazol-2-one derivative, hydrogen bonding

## Abstract

In the title compound, C_17_H_19_ClN_4_O_5_, the benzimidazole fused-ring system is essentially planar, the maximum deviation from the mean plane being 0.06 (1) Å. Both oxazolidine rings are nearly planar, the maximum deviations from the mean planes are 0.071 (13) and 0.070 (10) Å. The dihedral angle between the mean planes of the oxazolidine rings is 69.9 (7)°. The benzimidazole mean plane makes the dihedral angles of 43.9 (6) and 45.6 (6)° with the two oxazolidine rings. In the crystal, the mol­ecules are linked together by weak C—H⋯O hydrogen bonds building zigzag tapes running along the *c* axis. The Cl atom is split over two positions with an occupancy ratio of 0.567 (7):0.433 (7). This means that the reaction yields two isomers, *A* and *B*; the *A* component has the Cl-atom substituent in the 5-position of the benzimidazolone ring and the *B* component has the Cl atom in the 6-position. The two isomers form the disordered co-crystal, with a nearly half Cl atom in each of them, as indicated by the occupancy ratio. The crystal structure was refined as an inversion twin.

## Related literature   

For biological properties of benzimidazol-2-one derivatives, see: Gribkoff *et al.* (1994 ▸); Olesen *et al.* (1994 ▸); Soderlind *et al.* (1999 ▸). For anti­bacterial activity oxazolidin-2-ones, see: Diekema & Jones (2000 ▸); Mukhtar & Wright (2005 ▸). For asymmetric reactions of oxazolidin-2-ones, see: Evans *et al.* (1993 ▸); Matsunaga *et al.* (2005 ▸). For oxazolidin-2-one derivatives, see: Ouzidan *et al.* (2011 ▸); Dardouri *et al.* (2011 ▸).
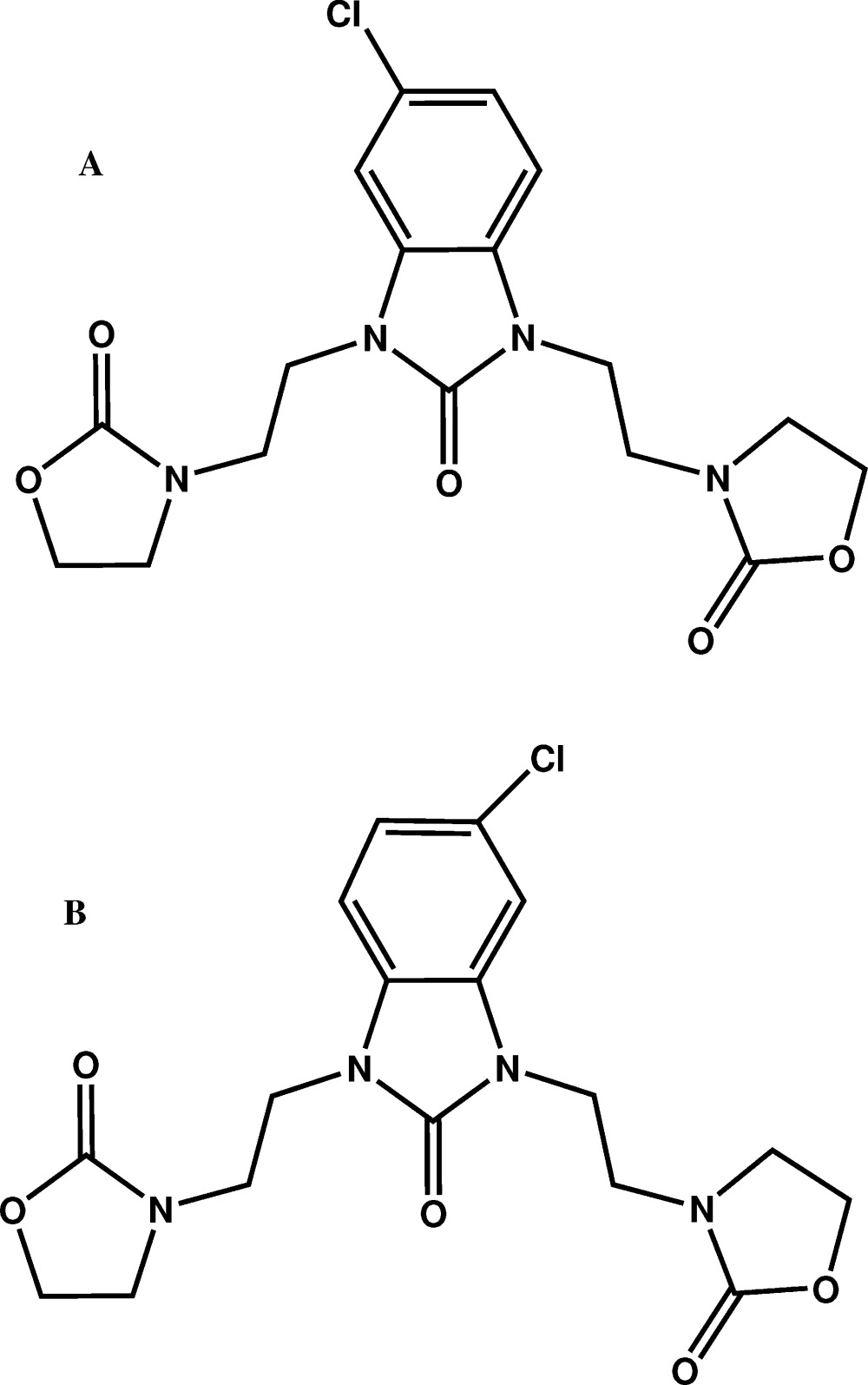


## Experimental   

### Crystal data  

C_17_H_19_ClN_4_O_5_
*M* *_r_* = 394.81
Orthorhombic, 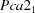

*a* = 14.053 (8) Å
*b* = 13.438 (6) Å
*c* = 9.733 (4) Å
*V* = 1838.1 (15) Å^3^
*Z* = 4
Mo *K*α radiation
μ = 0.25 mm^−1^
*T* = 296 K
0.35 × 0.31 × 0.26 mm

### Data collection  

Bruker X8 APEX diffractometer
Absorption correction: multi-scan (*SADABS*; Bruker, 2009 ▸) *T*_min_ = 0.504, *T*_max_ = 0.748
9588 measured reflections
3701 independent reflections
1697 reflections with *I* > 2σ(*I*)
*R*_int_ = 0.052

### Refinement  

*R*[*F* ^2^ > 2σ(*F* ^2^)] = 0.057
*wR*(*F* ^2^) = 0.150
*S* = 1.01
3701 reflections
255 parameters
4 restraints
H-atom parameters constrained
Δρ_max_ = 0.21 e Å^−3^
Δρ_min_ = −0.16 e Å^−3^
Absolute structure: Refined as an inversion twin
Absolute structure parameter: 0.5 (5)

### 

Data collection: *APEX2* (Bruker, 2009 ▸); cell refinement: *SAINT-Plus* (Bruker, 2009 ▸); data reduction: *SAINT-Plus* ; program(s) used to solve structure: *SHELXS97* (Sheldrick, 2008 ▸); program(s) used to refine structure: *SHELXL2013* (Sheldrick, 2015 ▸); molecular graphics: *ORTEP-3* (Burnett & Johnson, 1996 ▸) and *ORTEP-3 for Windows* (Farrugia, 2012 ▸); software used to prepare material for publication: *PLATON* (Spek, 2009 ▸) and *publCIF* (Westrip, 2010 ▸).

## Supplementary Material

Crystal structure: contains datablock(s) I, global. DOI: 10.1107/S2056989015016102/xu5863sup1.cif

Structure factors: contains datablock(s) I. DOI: 10.1107/S2056989015016102/xu5863Isup2.hkl

Supporting information file. DOI: 10.1107/S2056989015016102/xu5863Isup3.cml

**Fig. 1.**
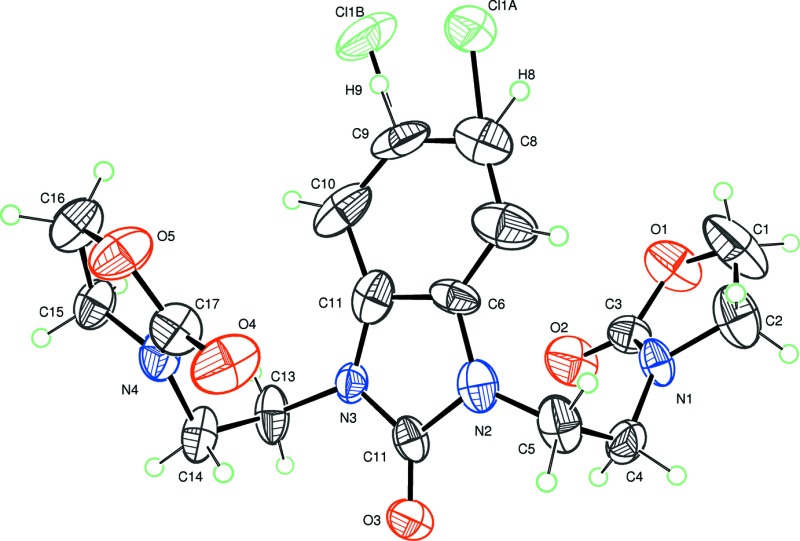
: Molecular plot the title compound with the atom-labelling scheme. Displacement ellipsoids are drawn at the 50% probability level. H atoms are represented as small circles.

**Fig. 2.**
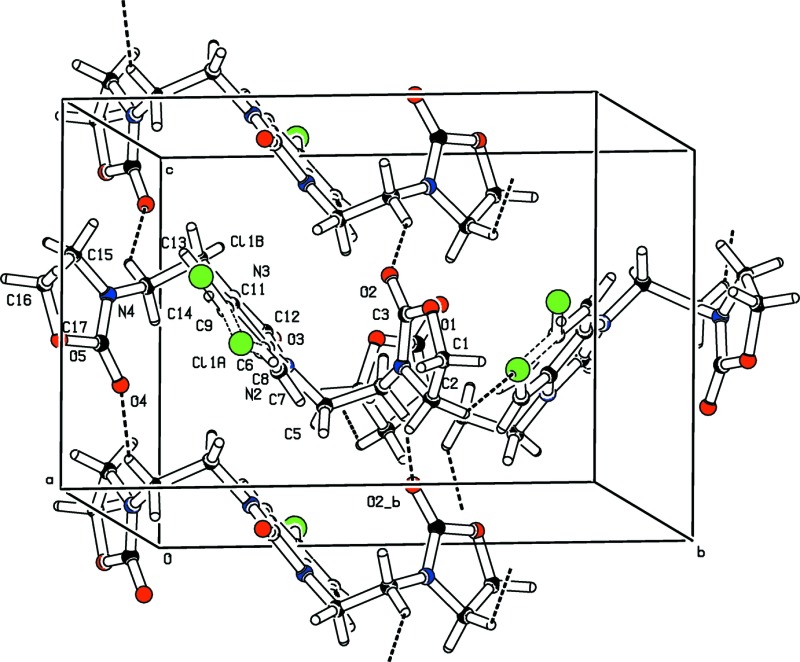
: Intermolecular interactions in the title compound building a zigzag tape along c axis. Hydrogen bonds are shown as dashed lines.

. DOI: 10.1107/S2056989015016102/xu5863fig1.tif
Mol­ecular plot the title compound with the atom-labelling scheme. Displacement ellipsoids are drawn at the 50% probability level. H atoms are represented as small circles.

c . DOI: 10.1107/S2056989015016102/xu5863fig2.tif
Inter­molecular inter­actions in the title compound building a zigzag tape along *c* axis. Hydrogen bonds are shown as dashed lines.

CCDC reference: 1421051

Additional supporting information:  crystallographic information; 3D view; checkCIF report

## Figures and Tables

**Table 1:** Hydrogen-bond geometry (, )

*D*H*A*	*D*H	H*A*	*D* *A*	*D*H*A*
C4H4*A*O2^i^	0.97	2.42	3.247(13)	143
C14H14*A*O4^ii^	0.97	2.48	3.315(13)	144

Symmetry codes: (i) 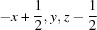; (ii) 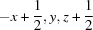.

## supplementary crystallographic information

### S1. Comment

Benzimidazol-2-one derivatives are useful heterocyclic building blocks and are prominent structural elements of compounds demonstrating a wide variety of pharmacological and biochemical properties(Gribkoff *et al.*, 1994; Olesen *et al.*, 1994; Soderlind *et al.*, 1999).

Also, oxazolidin-2-ones are a very important class of heterocyclic compounds and their derivatives have attracted attention in various areas of drug development for antibacterial activity (Diekema & Jones, 2000; Mukhtar & Wright, 2005). Some oxazolidin-2-ones have been used as chiral auxiliaries in a wide range of asymmetric reactions (Evans *et al.*, 1993; Matsunaga *et al.*, 2005). In a previous study, we reacted 1*H*-benzo[*d*]imidazol-2(3*H*)-one with bis(2-chloroethyl)amine hydrochloride in the presence of a catalytic quantity of tetra-n-butylammonium bromide to form 1,3-bis(2-(2-oxooxazolidin-3-yl)ethyl)-1*H*-benzo[*d*]imidazol-2(3*H*)-one (Ouzidan *et al.*, 2011). The study is extended to the synthesis of the 5-chloro analog to furnish the title compound (Scheme 1).

The molecule of title compound is build up from a fused five- and six-membered rings linked through ethyl groups, on opposite side, to two 2-oxo-oxazolidin- 3-yl rings as shown in Fig. 1. The chlorine in 5-chloro-benzo[*d*]imidazol-2(3*H*) -one is splited in two positions with an occupancy ratio of Cl1B = 0.567 (7) and Cl1A = 0.433 (7). As a matter of fact, we have two isomers that form a disordered co-crystal, like in the 5-Chloro-1-[(*E*)-3-(dimethylamino)acryloyl]- 3-methyl-1*H*-benzimidazol-2(3*H*)-one-6-chloro-1-[(*E*)-3-(dimethylamino)acryloyl]- 3-methyl-1*H*-benzimidazol-2(3*H*)-one(4/1) (Dardouri *et al.*, 2011), but with a nearly half chlorine atom in each of them as shown in the occupancy ratio of Cl1A and Cl1B. The fused rings system (N2N3C6 – C12) is essentially planar with the largest deviation from the mean plane being 0.06 (1) Å at C8 atom. The benzimidazole plane makes dihedral angles of 43.9 (6)° and 45.6 (6)° with the two 2-oxo-oxazolidin-3-yl rings, (O1N1C1-C3) and (O5N4C15-C17), respectively. The dihedral angle between the two 2-oxo-oxazolidin-3-yl rings is of 69.9 (7)°. In the crystal, the molecules are linked together by C4–H4A···O2 and C14–H14A···O4 hydrogen bonds in the way to build a zigzag tape along *c* axis as shown in Fig. 2 and Table 2.

### S2. Experimental

To 5-chloro-1*H*-benzo[*d*]imidazol-2(3*H*)-one (0,2 g, 1.18 mmol), potassium carbonate (0.65 g, 4.74 mmol), and tetra-n-butylammonium bromide (0.05 g, 0,1 mmol) in DMF (15 ml) was added bis(2-chloroethyl)amine hydrochloride (0.52 g, 3 mmol). The mixture was heated for 48 h. After the completion of the reaction (as monitored by TLC), the inorganic material salt was filtered and the solvent was removed under reduced pressure. The residue was purified by column chromatography on silica gel by using (ethanol/ethylacetate: 1/4) as eluent to furnish colourless crystals.

### S3. Refinement

Crystal data, data collection and structure refinement details are summarized in Table 1. In the molecule there is a pseudo center of symmetry but the results of the structure refinement in the centro symmetric *Pbcn* space group are not satisfactory. The absolute structure cannot be determined reliably and the structure is refined as a 2-component inversion twin.

All H atoms could be located in a difference Fourier map. However, they were placed in calculated positions with C—H = 0.93 Å (aromatic), and C—H = 0.97 Å (methylene) and refined as riding on their parent atoms with *U*_iso_(H) = 1.2 *U*_eq_ (C).

The chlorine atom is disordered over two positions so that leads to two isomers: 5-chloro-3-methylbenzimidazol-2-one component and a 6-chloro-3-methylbenzimidazol-2-one. The occupancy refined to an 0.567 (7): 0.433 (7) ratio. The C7–C8, C8–C9 and C9–C10 distances were restrained to 1.38 (1) Å.

### Crystal data

**Table:**

C_17_H_19_ClN_4_O_5_	*D*_x_ = 1.423 Mg m^−^^3^
*M**_r_* = 394.81	Mo *K*α radiation, λ = 0.71073 Å
Orthorhombic, *P**c**a*2_1_	Cell parameters from 3701 reflections
*a* = 14.053 (8) Å	θ = 1.5–26.4°
*b* = 13.438 (6) Å	µ = 0.25 mm^−^^1^
*c* = 9.733 (4) Å	*T* = 296 K
*V* = 1838.1 (15) Å^3^	Block, colourless
*Z* = 4	0.35 × 0.31 × 0.26 mm
*F*(000) = 820	

### Data collection

**Table:**

Bruker X8 APEX diffractometer	3701 independent reflections
Radiation source: fine-focus sealed tube	1697 reflections with *I* > 2σ(*I*)
Graphite monochromator	*R*_int_ = 0.052
φ and ω scans	θ_max_ = 26.4°, θ_min_ = 1.5°
Absorption correction: multi-scan (*SADABS*; Bruker, 2009)	*h* = −13→17
*T*_min_ = 0.504, *T*_max_ = 0.748	*k* = −16→16
9588 measured reflections	*l* = −12→12

### Refinement

**Table:**

Refinement on *F*^2^	Hydrogen site location: inferred from neighbouring sites
Least-squares matrix: full	H-atom parameters constrained
*R*[*F*^2^ > 2σ(*F*^2^)] = 0.057	*w* = 1/[σ^2^(*F*_o_^2^) + (0.0473*P*)^2^ + 0.3226*P*] where *P* = (*F*_o_^2^ + 2*F*_c_^2^)/3
*wR*(*F*^2^) = 0.150	(Δ/σ)_max_ < 0.001
*S* = 1.01	Δρ_max_ = 0.21 e Å^−^^3^
3701 reflections	Δρ_min_ = −0.16 e Å^−^^3^
255 parameters	Absolute structure: Refined as an inversion twin.
4 restraints	Absolute structure parameter: 0.5 (5)

### Special details

**Table:**

Geometry. All e.s.d.'s (except the e.s.d. in the dihedral angle between two l.s. planes) are estimated using the full covariance matrix. The cell e.s.d.'s are taken into account individually in the estimation of e.s.d.'s in distances, angles and torsion angles; correlations between e.s.d.'s in cell parameters are only used when they are defined by crystal symmetry. An approximate (isotropic) treatment of cell e.s.d.'s is used for estimating e.s.d.'s involving l.s. planes.
Refinement. Refined as a 2-component inversion twin.

### Fractional atomic coordinates and isotropic or equivalent isotropic displacement parameters (Å^2^)

**Table:**

	*x*	*y*	*z*	*U*_iso_*/*U*_eq_	Occ. (<1)
C1	0.5061 (11)	0.6238 (11)	0.3946 (17)	0.141 (6)	
H1A	0.5660	0.5943	0.3660	0.169*	
H1B	0.5119	0.6955	0.3879	0.169*	
C2	0.4284 (10)	0.5885 (9)	0.3051 (13)	0.094 (4)	
H2A	0.3888	0.6431	0.2737	0.113*	
H2B	0.4527	0.5523	0.2264	0.113*	
C3	0.4066 (8)	0.5371 (8)	0.5275 (13)	0.060 (3)	
C4	0.2918 (6)	0.4698 (6)	0.3603 (12)	0.068 (3)	
H4A	0.2585	0.5066	0.2894	0.082*	
H4B	0.2501	0.4650	0.4395	0.082*	
C5	0.3133 (7)	0.3677 (7)	0.3089 (10)	0.074 (3)	
H5A	0.2552	0.3394	0.2721	0.089*	
H5B	0.3582	0.3733	0.2336	0.089*	
C6	0.4456 (8)	0.2814 (7)	0.4386 (11)	0.058 (3)	
C7	0.5213 (9)	0.3189 (9)	0.3704 (15)	0.093 (4)	
H7	0.5100	0.3570	0.2923	0.111*	
C8	0.6121 (8)	0.3041 (8)	0.4091 (11)	0.093 (3)	
H8	0.6648	0.3351	0.3704	0.112*	0.567 (7)
C9	0.6151 (7)	0.2357 (7)	0.5151 (15)	0.085 (4)	
H9	0.6762	0.2161	0.5395	0.102*	0.433 (7)
C10	0.5401 (8)	0.1888 (8)	0.5959 (13)	0.087 (4)	
H10	0.5521	0.1462	0.6692	0.104*	
C11	0.4446 (10)	0.2151 (7)	0.5506 (11)	0.067 (3)	
C12	0.2912 (5)	0.2497 (9)	0.5031 (13)	0.0513 (14)	
C13	0.3124 (7)	0.1304 (6)	0.6914 (9)	0.071 (3)	
H13A	0.2538	0.1570	0.7293	0.085*	
H13B	0.3582	0.1240	0.7654	0.085*	
C14	0.2935 (7)	0.0280 (6)	0.6264 (11)	0.067 (3)	
H14A	0.2558	−0.0113	0.6898	0.080*	
H14B	0.2560	0.0373	0.5437	0.080*	
C15	0.4330 (8)	−0.0839 (8)	0.6883 (11)	0.068 (3)	
H15A	0.3959	−0.1385	0.7256	0.081*	
H15B	0.4552	−0.0423	0.7632	0.081*	
C16	0.5157 (7)	−0.1221 (8)	0.6022 (10)	0.075 (3)	
H16A	0.5743	−0.0876	0.6247	0.090*	
H16B	0.5248	−0.1931	0.6146	0.090*	
C17	0.4082 (9)	−0.0368 (8)	0.4637 (11)	0.066 (3)	
N1	0.3770 (6)	0.5234 (5)	0.3986 (8)	0.052 (2)	
N2	0.3510 (7)	0.3007 (6)	0.4064 (9)	0.064 (2)	
N3	0.3503 (6)	0.1993 (5)	0.5834 (8)	0.0473 (19)	
N4	0.3790 (6)	−0.0276 (6)	0.5914 (9)	0.059 (2)	
O1	0.4858 (6)	0.5965 (7)	0.5304 (9)	0.094 (3)	
O2	0.3747 (6)	0.4989 (7)	0.6326 (8)	0.094 (3)	
O3	0.2045 (3)	0.2489 (6)	0.4997 (11)	0.0705 (11)	
O4	0.3742 (6)	−0.0045 (6)	0.3617 (8)	0.096 (3)	
O5	0.4846 (6)	−0.0991 (6)	0.4629 (8)	0.087 (3)	
Cl1A	0.7305 (5)	0.2867 (5)	0.4090 (11)	0.103 (4)	0.433 (7)
Cl1B	0.7313 (5)	0.2114 (5)	0.5813 (11)	0.140 (4)	0.567 (7)

### Atomic displacement parameters (Å^2^)

**Table:**

	*U*^11^	*U*^22^	*U*^33^	*U*^12^	*U*^13^	*U*^23^
C1	0.139 (13)	0.160 (14)	0.123 (13)	−0.094 (11)	0.019 (12)	0.004 (12)
C2	0.131 (13)	0.087 (10)	0.064 (8)	−0.026 (9)	0.012 (9)	0.007 (7)
C3	0.053 (8)	0.066 (7)	0.060 (7)	−0.007 (5)	−0.007 (6)	−0.006 (6)
C4	0.067 (9)	0.056 (7)	0.082 (8)	−0.004 (5)	−0.033 (6)	0.012 (6)
C5	0.104 (10)	0.066 (8)	0.053 (6)	−0.031 (6)	−0.025 (7)	0.003 (6)
C6	0.040 (7)	0.060 (6)	0.074 (8)	−0.013 (6)	0.008 (6)	−0.028 (6)
C7	0.070 (8)	0.105 (9)	0.103 (8)	−0.019 (7)	0.012 (7)	−0.040 (7)
C8	0.076 (8)	0.106 (9)	0.097 (7)	−0.015 (6)	0.012 (6)	−0.010 (6)
C9	0.047 (5)	0.071 (8)	0.137 (11)	0.024 (5)	−0.028 (8)	−0.047 (7)
C10	0.099 (10)	0.077 (7)	0.084 (7)	0.048 (8)	−0.036 (7)	−0.051 (6)
C11	0.099 (11)	0.044 (5)	0.058 (7)	0.009 (6)	−0.012 (7)	−0.020 (6)
C12	0.070 (4)	0.041 (3)	0.043 (3)	−0.013 (7)	0.002 (7)	−0.003 (2)
C13	0.120 (11)	0.045 (6)	0.047 (5)	0.003 (6)	0.021 (6)	0.008 (5)
C14	0.102 (10)	0.046 (6)	0.052 (6)	0.018 (6)	0.018 (6)	0.007 (5)
C15	0.083 (9)	0.063 (8)	0.056 (6)	0.014 (6)	−0.006 (6)	0.013 (6)
C16	0.089 (8)	0.082 (7)	0.055 (6)	0.023 (6)	−0.013 (6)	−0.007 (6)
C17	0.079 (10)	0.080 (8)	0.038 (6)	0.000 (7)	0.006 (7)	−0.003 (6)
N1	0.075 (6)	0.037 (5)	0.042 (4)	−0.019 (4)	0.000 (5)	−0.004 (4)
N2	0.090 (7)	0.048 (5)	0.055 (5)	−0.005 (5)	0.005 (6)	−0.007 (4)
N3	0.054 (5)	0.041 (5)	0.047 (4)	0.003 (4)	0.001 (4)	0.010 (4)
N4	0.079 (7)	0.059 (6)	0.040 (4)	0.004 (5)	0.015 (5)	0.013 (4)
O1	0.096 (7)	0.120 (7)	0.064 (5)	−0.036 (6)	−0.001 (5)	−0.019 (5)
O2	0.097 (7)	0.135 (7)	0.051 (5)	−0.013 (5)	0.005 (4)	0.012 (5)
O3	0.055 (3)	0.068 (2)	0.090 (3)	−0.008 (5)	0.002 (7)	−0.0005 (19)
O4	0.098 (7)	0.151 (8)	0.038 (4)	0.031 (5)	0.003 (4)	0.019 (5)
O5	0.082 (6)	0.113 (7)	0.066 (5)	0.037 (5)	0.015 (5)	−0.008 (5)
Cl1A	0.067 (6)	0.075 (5)	0.166 (8)	−0.006 (4)	0.006 (5)	0.010 (5)
Cl1B	0.071 (5)	0.123 (5)	0.226 (9)	0.038 (4)	−0.040 (5)	0.020 (6)

### Geometric parameters (Å, º)

**Table:**

C1—O1	1.401 (15)	C9—Cl1A	2.042 (15)
C1—C2	1.475 (17)	C9—H9	0.9300
C1—H1A	0.9700	C10—C11	1.456 (15)
C1—H1B	0.9700	C10—H10	0.9300
C2—N1	1.454 (13)	C11—N3	1.379 (14)
C2—H2A	0.9700	C12—O3	1.219 (6)
C2—H2B	0.9700	C12—N3	1.326 (12)
C3—O2	1.230 (14)	C12—N2	1.436 (13)
C3—N1	1.334 (14)	C13—N3	1.499 (11)
C3—O1	1.369 (12)	C13—C14	1.538 (12)
C4—N1	1.447 (11)	C13—H13A	0.9700
C4—C5	1.491 (12)	C13—H13B	0.9700
C4—H4A	0.9700	C14—N4	1.455 (11)
C4—H4B	0.9700	C14—H14A	0.9700
C5—N2	1.411 (12)	C14—H14B	0.9700
C5—H5A	0.9700	C15—N4	1.428 (12)
C5—H5B	0.9700	C15—C16	1.522 (13)
C6—C7	1.352 (16)	C15—H15A	0.9700
C6—N2	1.391 (13)	C15—H15B	0.9700
C6—C11	1.408 (8)	C16—O5	1.458 (11)
C7—C8	1.345 (10)	C16—H16A	0.9700
C7—H7	0.9300	C16—H16B	0.9700
C8—C9	1.383 (11)	C17—O4	1.183 (14)
C8—Cl1A	1.680 (13)	C17—N4	1.315 (14)
C8—H8	0.9300	C17—O5	1.362 (13)
C9—C10	1.458 (10)	Cl1A—Cl1B	1.959 (6)
C9—Cl1B	1.786 (10)		
			
O1—C1—C2	108.8 (11)	N3—C11—C6	106.6 (12)
O1—C1—H1A	109.9	N3—C11—C10	141.1 (11)
C2—C1—H1A	109.9	C6—C11—C10	112.2 (14)
O1—C1—H1B	109.9	O3—C12—N3	129.7 (12)
C2—C1—H1B	109.9	O3—C12—N2	124.8 (12)
H1A—C1—H1B	108.3	N3—C12—N2	105.3 (5)
N1—C2—C1	101.1 (10)	N3—C13—C14	109.0 (7)
N1—C2—H2A	111.6	N3—C13—H13A	109.9
C1—C2—H2A	111.6	C14—C13—H13A	109.9
N1—C2—H2B	111.6	N3—C13—H13B	109.9
C1—C2—H2B	111.6	C14—C13—H13B	109.9
H2A—C2—H2B	109.4	H13A—C13—H13B	108.3
O2—C3—N1	127.6 (10)	N4—C14—C13	114.4 (8)
O2—C3—O1	121.5 (11)	N4—C14—H14A	108.7
N1—C3—O1	110.7 (11)	C13—C14—H14A	108.7
N1—C4—C5	112.2 (8)	N4—C14—H14B	108.7
N1—C4—H4A	109.2	C13—C14—H14B	108.7
C5—C4—H4A	109.2	H14A—C14—H14B	107.6
N1—C4—H4B	109.2	N4—C15—C16	102.8 (8)
C5—C4—H4B	109.2	N4—C15—H15A	111.2
H4A—C4—H4B	107.9	C16—C15—H15A	111.2
N2—C5—C4	115.9 (8)	N4—C15—H15B	111.2
N2—C5—H5A	108.3	C16—C15—H15B	111.2
C4—C5—H5A	108.3	H15A—C15—H15B	109.1
N2—C5—H5B	108.3	O5—C16—C15	102.2 (8)
C4—C5—H5B	108.3	O5—C16—H16A	111.3
H5A—C5—H5B	107.4	C15—C16—H16A	111.3
C7—C6—N2	124.9 (12)	O5—C16—H16B	111.3
C7—C6—C11	128.7 (15)	C15—C16—H16B	111.3
N2—C6—C11	106.4 (12)	H16A—C16—H16B	109.2
C8—C7—C6	123.6 (14)	O4—C17—N4	129.3 (12)
C8—C7—H7	118.2	O4—C17—O5	122.5 (11)
C6—C7—H7	118.2	N4—C17—O5	108.0 (10)
C7—C8—C9	109.6 (11)	C3—N1—C4	124.7 (9)
C7—C8—Cl1A	163.7 (10)	C3—N1—C2	110.5 (9)
C9—C8—Cl1A	83.0 (9)	C4—N1—C2	123.4 (9)
C7—C8—H8	125.2	C6—N2—C5	129.0 (10)
C9—C8—H8	125.2	C6—N2—C12	108.8 (9)
Cl1A—C8—H8	43.4	C5—N2—C12	121.7 (10)
C8—C9—C10	131.9 (10)	C12—N3—C11	112.8 (8)
C8—C9—Cl1B	114.8 (10)	C12—N3—C13	120.4 (9)
C10—C9—Cl1B	112.8 (9)	C11—N3—C13	126.7 (9)
C8—C9—Cl1A	54.8 (8)	C17—N4—C15	114.1 (10)
C10—C9—Cl1A	172.8 (7)	C17—N4—C14	121.8 (10)
Cl1B—C9—Cl1A	61.2 (4)	C15—N4—C14	123.8 (8)
C8—C9—H9	114.0	C3—O1—C1	107.4 (10)
C10—C9—H9	114.0	C17—O5—C16	111.2 (8)
Cl1B—C9—H9	8.4	C8—Cl1A—Cl1B	94.4 (5)
Cl1A—C9—H9	59.3	C8—Cl1A—C9	42.2 (4)
C11—C10—C9	113.4 (10)	Cl1B—Cl1A—C9	53.0 (4)
C11—C10—H10	123.3	C9—Cl1B—Cl1A	65.9 (6)
C9—C10—H10	123.3		

### Hydrogen-bond geometry (Å, º)

**Table:**

*D*—H···*A*	*D*—H	H···*A*	*D*···*A*	*D*—H···*A*
C4—H4*A*···O2^i^	0.97	2.42	3.247 (13)	143
C14—H14*A*···O4^ii^	0.97	2.48	3.315 (13)	144

Symmetry codes: (i) −*x*+1/2, *y*, *z*−1/2; (ii) −*x*+1/2, *y*, *z*+1/2.

## References

[bb1] Bruker (2009). *APEX2*, *SAINT-Plus* and *SADABS*. Bruker AXS Inc., Madison, Wisconsin, USA.

[bb2] Burnett, M. N. & Johnson, C. K. (1996). *ORTEPIII*. Report ORNL-6895. Oak Ridge National Laboratory, Tennessee, USA.

[bb3] Dardouri, R., Rodi, Y. K., Saffon, N., Essassi, E. M. & Ng, S. W. (2011). *Acta Cryst.* E**67**, o1853. 10.1107/S1600536811024706 PMC3151851 21837218

[bb4] Diekema, D. J. & Jones, R. N. (2000). *Drugs*, **59**, 7–16. 10.2165/00003495-200059010-00002 10718097

[bb5] Evans, D. A., Ng, H. P. & Rieger, D. L. (1993). *J. Am. Chem. Soc.* **115**, 11446–11459.

[bb6] Farrugia, L. J. (2012). *J. Appl. Cryst.* **45**, 849–854.

[bb7] Gribkoff, V. K., Champigny, G., Barbry, P., Dworetzky, S. I., Meanwell, N. A. & Lazdunski, M. (1994). *J. Biol. Chem.* **269**, 10983–10986. 7512555

[bb8] Matsunaga, H., Ishizuka, T. & Kunieda, T. (2005). *Tetrahedron*, **61**, 8073–8094.

[bb9] Mukhtar, T. A. & Wright, G. D. (2005). *Chem. Rev.* **105**, 529–542. 10.1021/cr030110z 15700955

[bb10] Olesen, S. P., Munch, E., Moldt, P. & Drejer, J. (1994). *Eur. J. Pharmacol.* **251**, 53–59. 10.1016/0014-2999(94)90442-1 8137869

[bb11] Ouzidan, Y., Kandri Rodi, Y., Fronczek, F. R., Venkatraman, R., El Ammari, L. & Essassi, E. M. (2011). *Acta Cryst.* E**67**, o362–o363. 10.1107/S1600536810052141 PMC3051530 21523041

[bb12] Sheldrick, G. M. (2008). *Acta Cryst.* A**64**, 112–122. 10.1107/S0108767307043930 18156677

[bb13] Sheldrick, G. M. (2015). *Acta Cryst.* C**71**, 3–8.

[bb14] Soderlind, K. J., Gorodetsky, B., Singh, A. K., Bachur, N., Miller, G. G. & Lown, J. W. (1999). *Anticancer Drug. Des.* **14**, 19–36. 10363025

[bb15] Spek, A. L. (2009). *Acta Cryst.* D**65**, 148–155. 10.1107/S090744490804362X PMC2631630 19171970

[bb16] Westrip, S. P. (2010). *J. Appl. Cryst.* **43**, 920–925.

