# Supplementary figures and images for: Crystal structure of 5-chloro-1,3-bis­[2-(2-oxo-1,3-oxazolidin-3-yl)eth­yl]-1H-benz­imidazol-2(3H)-one
Source: Acta Crystallogr E Crystallogr Commun. 2015 Sep 12;71(Pt 10):o735–6. doi: 10.1107/S2056989015016102 (PMC4647354; doi:10.1107/S2056989015016102)

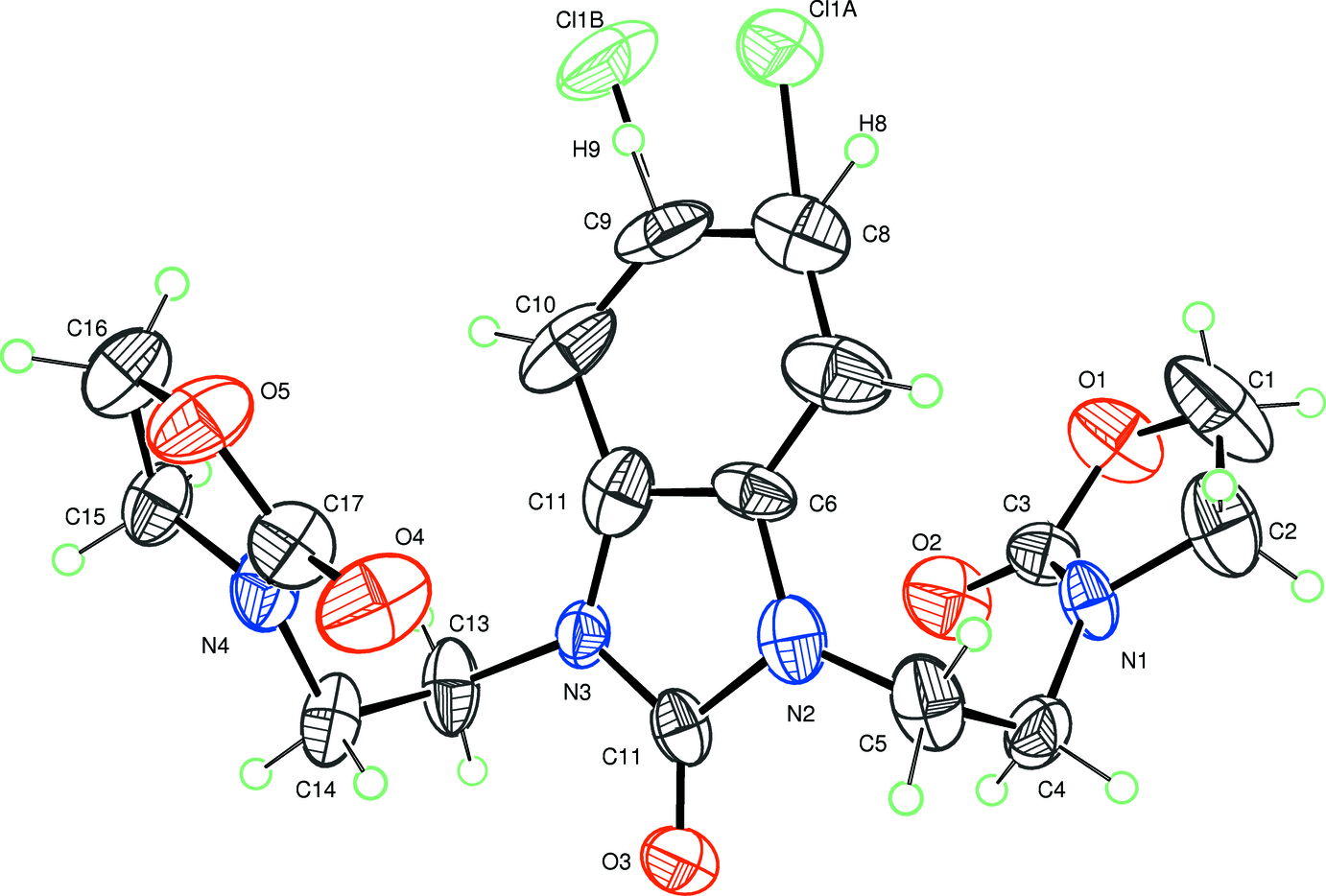

Supplement: Supplementary file 4 [file e-71-0o735-fig1.tif]

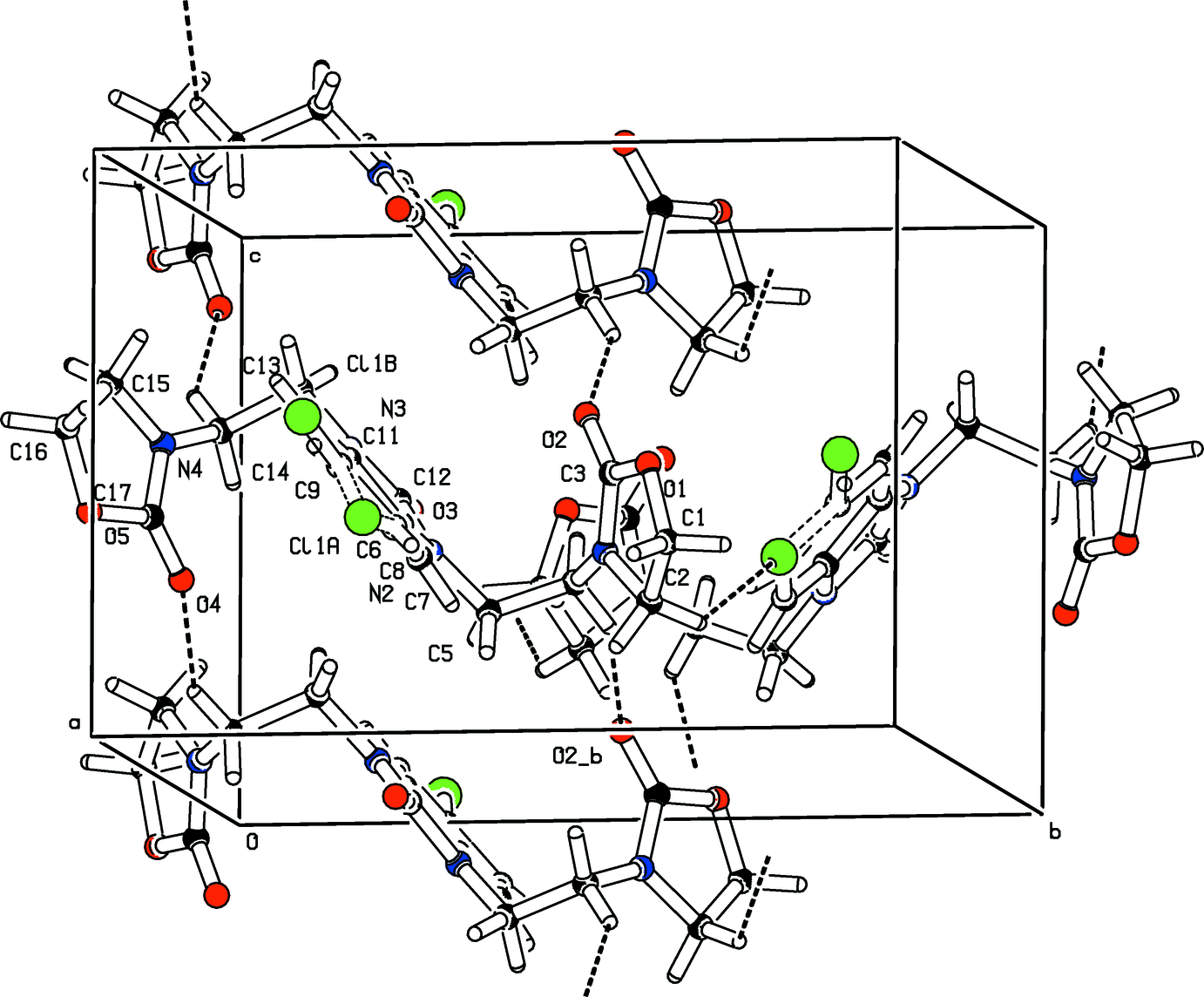

Supplement: Supplementary file 5 [file e-71-0o735-fig2.tif]
